# Changing methodology results in operational drift in the meaning of leaf area index, necessitating implementation of foliage layer index

**DOI:** 10.1002/ece3.3662

**Published:** 2017-12-03

**Authors:** Gillian L. Rapson

**Affiliations:** ^1^ Ecology Group College of Science Massey University Palmerston North New Zealand

**Keywords:** agriculture, chlorophyll, cover repetition, growth analysis, leaf area, shade‐light

## Abstract

Leaf area index (LAI) was developed to describe the number of layers of foliage in a monoculture. Subsequent expansion into measurement by remote‐sensing methods has resulted in misrepresentation of LAI. The new name foliage layer index (FLI) is applied to a more simply estimated version of Goodall's “cover repetition,” that is, the number of layers of foliage a single species has, either within a community or in monoculture. The relationship of FLI with cover is demonstrated in model communities, and some potential relationships between FLI and species’ habit are suggested. FLI_comm_ is a new formulation for the number of layers of foliage in a mixed‐species’ community. LAI should now be reserved for remote‐sensing applications in mixed communities, where it is probably a nonlinear measure of the density of light‐absorbing pigments.

## 1 INTRODUCTION

Leaf area index (LAI) has been widely adopted today, rating over 259,000 mentions in Google Scholar, while there are at least 1,000 entries to the global LAI database (Asner, Scurlock, & Hicke, 2003) and 2606 records for woody species in the meta‐analysis of Iio, Hikosaka, Anten, Nakagawa, and Ito (2014). LAI is one of the earliest of the growth analysis variates, originally developed by agronomists to study the performance of crops in the field. The term “growth analysis variate” refers to an extensive family of quantitative metrics exploring how a plant grows. The growth analysis monograph is Clifford Evan's book on *The quantitative analysis of plant growth* (Evans, 1972; perhaps the family of plant growth analysis variates should be called by the honorific of “Evans’ indices”?). A handy summary is available in the “Studies in Biology” series on *Plant growth analysis* by Roderick Hunt (Hunt, 1978). Growth analysis variates are all based on assimilate partitioning strategies—how a plant allocates the carbohydrates it has available for growth. They include those which measure simple, one‐time, plant responses such as height and shoot/root ratio, Leaf Area Ratio (LAR; amount of leaf area per unit dry weight of the plant), and specific leaf area (SLA; amount of leaf surface per unit leaf weight), this last now widely used in trait research (Díaz et al., 2016; Freschet, Swart, & Cornelissen, 2015; Vile et al., 2005). More complex variates include those specifically designed to be computed over periods of time, such as the rate at which a plant grows (Relative Growth Rate; RGR), its rate of assimilation (Net Assimilation Rate, NAR a.k.a. ULR or Unit Leaf Rate), and measures of the relative rate at which a plant allocates assimilate to its various tissues, such as RGR (shoot)/RGR (root). Although change over time can also be calculated for LAI, it is commonly derived for a single measurement time.

Leaf area index reports one aspect, that is, layering, of the way a plant places its foliage, which is crucial in its interception of light for photosynthesis, and hence its overall productivity. Two general approaches to the derivation of LAIs are now apparent in the literature. The first uses actual measurements of leaf area (e.g., Camargo et al., 2016; Odum, Copeland, & Brown, 1963; Njuguna, Kamiri, Okalebo, Ngetich, & Kebeney, 2016; Zaman, Karim, Bari, Akter, & Ahmed, 2016). The second, of more recent origin, uses proxy measurements, such as light‐sensing, hand‐held scanners or remote‐sensing satellites (e.g., Clevers, Kooistra, & van den Brande, 2017; Kim et al., 2017; Verger, Filella, Baret, & Peñuelas, 2016). Thus, as methodology has developed, LAI's meaning has deviated from the original definition. This operational drift is often unheeded or unknown by its users, leading to potential misinterpretations in its application, and so requires clarification and repair.

## 2 What is LAI?

Leaf area index or LAI originated in a paper on comparative physiology of crop growth (Watson, 1947), thusly:

“.. the measure of leaf area which is relevant to the comparison of agricultural yields, that is, of weights of different crops produced per unit area of land, is the leaf area per unit area of land, which it is proposed to call the Leaf Area Index (LAI). A value of 2 for LAI, for example, indicates that there were 2 acres of leaf surface on an acre of crop… LAI at the time of maximum leaf area was of the same order of magnitude for [different] crops, ranging from 2 to 4.”

Thus, the equation for LAI can be given as:

$$\text{LAI}\;=\;\frac{\text{total leaf area of a species}}{\text{ground area}}.$$

Wilson (2011), in a review of ways of measuring cover‐related variates, defined it as “leaf area of species **S** per unit area of ground,” while, in a remote‐sensing examination of LAI in woody species, Iio et al. (2014) defined LAI (which most authors do not), as “the amount of leaf area per unit ground area.” For the global database, presumably independent of measurement technique, Asner et al. (2003) defined LAI as “the amount of leaf area (m^2^) in a canopy per unit ground area (m^2^).” These are equivalent to the definition of Watson (1947), so the original definition is still the one widely reported today. But are the two approaches to measurement actually recording the same thing?

A farmer cares how much land is needed to produce a certain foliage area (and therefore biomass) of the planted crop, and usually that production is in a context where the crop species can occupy all the resources of the land “at will”; that is, the farmer is dealing with a monoculture. Now Watson (1947) clearly wished to have a variate which reported the number of layers of foliage which the crop (a single species) had produced. This was also Evan's interpretation (1972); p. 218), describing LAI as “obviously the average number of complete layers of leaves produced by the plant.”

A fundamental part of LAI is the need to measure leaf area. The original and usually destructive methods involve removing foliage and either estimating its area in some way (such as x.length.width) or using a cut‐and‐weigh technique or measuring biomass and converting that via SLA. Today leaf area is simply measured using photo‐metric methods that are so labor‐unintensive, rapid, and even, if wished, nondestructive, that it is hard to believe there were ever times when measuring leaf area was a chore and a challenge—see Goodall (1952) and Evans (1972) for views of the complexity of that process. Over the period 1947–1960, and partially later, the original meaning of LAI, involving direct measurements of leaf area, was applied for crops and almost always for monocultures (e.g., Brougham, 1960).

In about the 1970s another tool emerged on the scene which appeared to obviate this need to measure leaf area. Portable photogrammetric systems measure light attenuation through the canopy to estimate LAI at a point (e.g., Jordan, 1969). A great boon to crop scientists they were readily adopted. Expanding astronomically from the 1990s, remote‐sensing or satellite‐based methods measured spectral reflectance off foliage to estimate LAI, usually after complex scaling of the scanned wavelengths (e.g., Chen & Cihlar, 1996; Clevers et al., 2017; Kim et al., 2017; Verger et al., 2016). However, with these new tools, a change in the meaning and application of LAI has occurred.

## 3 OPERATIONAL DRIFT IN LAI

Evans (1972) talked about crops in a reasonably dense agricultural planting, and he specifically noted that their maximum LAI may vary greatly, graphing values of LAI up to 3. Recently, in a meta‐analysis of crops, Kang et al. (2016) recorded very few values above 5, with an average of 2.5. However, Asner et al. (2003. e.g., Fig. 5), compiling the world‐wide database of LAIs, graphed acceptable LAI scores of up to 10, though rejecting higher values. Iio et al. (2014) reported values up to and even over 20 for coniferous communities (usually with awl‐shaped leaves), while broadleaved communities (which tend to have bifacial leaves) had LAI values as high as about 12. What might such large LAI values mean?

Assuming there are no gaps in an upper monolayer, such as a canopy, then all lower layers must be photosynthesizing with radiation which has penetrated through the individual leaves of the canopy layer, and thus will be attenuated in photosynthetically active wavelengths. There will be a lower limit to the functionality of repeatedly attenuated radiation, influenced by the pigmentation, leaf angle, and specific leaf area of the species concerned (e.g., Vile et al., 2005; Blackburn, 2006; Díaz et al., 2016; Aneece, Epstein, & Lerdau, 2017). That there could be, say, 10 layers of foliage above the ground across the whole of a community is incredible, even in the most dense tropical forest. Considering leaves are usually small, and there are often gaps between them on a single plane, and if those gaps are a modest 50% of the horizontal space in any one of those 10 planes, then the “nongaps” must have 20 layers of leaves above a single point on the ground for LAI to average 10. With so many full layers of foliage above the ground layer in all directions, it is hard to see even sunflecks contributing much to daily photosynthesis in the lower layers (Way & Pearcy, 2012). Despite the occasional explanations of authors (e.g., Iio et al., 2014), their LAI values are unlikely to be directly interpretable as the physical number of layers of leaves.

Could other materials be inflating the LAIs reported? LAI uses “leaf” area because Watson and Evans were normally dealing with crops, which are generally annual and leafy. However, the portion measured is generally just the lamina, in fact the PSU (Photosynthetic Unit) of Smith et al. (1994), although this is seldom reported on. Other non‐laminal materials such as petioles and stems or even wood are omitted or factored in (or sometimes out) often without comment, but can be recorded as well (Bréda, 2003; Wilson, 2011). But most non‐laminal parts of a plant are such a small proportion of the volume (c.f. biomass) of any community (Chiarucci et al., in prep.), even in forest, that they are hardly worth considering in this way, while there are other dendrological tools for reporting quantities of woody materials (e.g., Bréda, 2003; Redpath & Rapson, 2015). Instead remotely sensed LAI, because it measures reflectance of radiation from an area of vegetation, is probably reporting some nonlinear aspect of the density of pigments absorbing in the photosynthetically active range, extending into the infrared (300–800 nm; e.g., Blackburn, 2006; Aneece et al., 2017). Pigment types and levels vary with species (e.g., Hughes & Smith, 2007; Zhu, Zhang, Zhang, & Peng, 2016), and, within individuals, with position in the canopy (e.g., Scartazza, Di Baccio, Bertolotto, Gavrichkova, & Matteucci, 2016), while their density varies with leaf thickness within a single foliage layer (Kume, 2017). Further, LAI values are probably being inflated due to some leaves not being held at right angles to the sensor (so that the path for light transmission through the leaf is longer than minimal), a scalar which likely increases in importance at higher LAI and for taller vegetation types. At the same time, they are probably being deflated by pigmented areas being “shaded” by reflective materials such as wood while being augmented by that scattered radiation. Thus, remotely‐sensed LAI is not directly interpretable as the number of layers of foliage.

Does LAI apply to a community? Watson (1947) and his successors worked on monocultures, where all leafy layers of a particular species would have only plastic variation in leaf placement and anatomy, even if differing in detail of the mesophyll, leaf size, and SLA, so that all layers of foliage would respond to the incident light regime within the same set of developmental constraints. But shading between different species elicits genetic as well as plastic adaptations. When both species and layers differ in their spectral consumption, then more reflected signal does not linearly mean more layers. If LAI is being used to report the number of foliage layers in a community, then ground‐truthing is required to define its relationship with the remotely sensed signal. Such standardization is probably routine when using under‐canopy scanners, but is seldom employed in remotely sensed LAI applications.

Leaf area index by remote sensing etc. has another significant feature here—it is dimensionless by methodology, working on what is effectively an infinitely small point of ground. Because LAI was originally developed for single‐species “vegetation,” it did return the number of layers of foliage of that species, because there was only one crop and it honestly did not matter whether the crop covered 1 m^2^ or 1 ha or a vanishingly small (but average) point. The crop was the same everywhere, and the same answer was obtained, regardless. In fact, remote sensing does produce plausible LAIs for monocultures (e.g., Kang et al., 2016). However, multispecies’ communities themselves cannot be dimensionless, individual species inevitably occupying interdigitating mosaics of the volume between the canopy and the ground. While an average signal can be obtained for a community, that average does not necessarily have any physical expression in terms of the vertical or tiered arrangement of the foliage of plant species within that community, such as might be measurable on the ground.

It seems too late to attempt to rename LAI used in remote‐sensing technologies (as should happen on the basis of seniority) so that it has some logical meaning in community‐deep assessments (whatever that meaning is). Instead, LAI should be released from its duties with respect to individual plants or species, which then require a new basis of expression for the number of their leafy layers.

## 4 FOLIAGE LAYER INDEX

Applying LAI as given by Watson (1947) to single species within a community gives an answer entirely dependent on the relative sizes of both the species being measured and the plot being sampled. It is conceivable that say an individual of the chosen species may occupy only half the plot, and so return an LAI<1. But if the plot is decreased in size so that same individual occupies all of the plot, then the LAI must be ≥1, all without the researcher doing anything other than following the standard methodology, and so probably without awareness of the issue.

Evans (1972; p. 218) was aware that LAI might be applied to a multispecies’ community and defined LAI per individual plant or species a little differently, as needing “the area of ground per plant, N^−1^[; *t*]he leaf area divided by this area of ground [(*N*)] gives the leaf area index (LAI).” Hunt (1978, p. 27) also notes that LAI can be calculated by multiplying plant leaf area per plant by the plant density, although this presupposes no bare ground. In this formulation, LAI cannot have values less than 1 as a plant must have some leaf area and some density. This contrasts with Watson's (1947) equation where the interpretation of LAI < 1 is that the crop is not (yet) covering all of the land surface which is available, which of course makes no sense for a single species within a community.

Naturally, Goodall (1952) noticed this too and derived a suitable index to measure foliage layers in such a community. He called it by the applicable, if rather vague term of “cover repetition” and defined it as “the number of times each pin hits the species while moving downward through the vegetation,” which Wilson (2011) interpreted thusly:

$$\frac{\text{Number of hits of the species}\;S}{\text{Number of pins with}\geq 1\;\text{hits of species}\;S.}$$

As a proxy method for measuring leaf area, lowering fine pins through the vegetation, and counting the number of times a pin touches a plant is a long, painful, and tedious method, although it can generate very reliable and credible results (e.g., Walker, Mark, & Wilson, 1995). Recommended when looking nondestructively for small or subtle differences, many ecologists might prefer less accurate, but more easily obtainable data, and replace quality with quantity, which is often as good, if not better, statistically.

Equivalent to the ratio of Fehmi's (2010) “leaf cover” and “aerial cover,” Wilson (2011) went on to give the ecological interpretation of his formulation as “Mean number of layers of leaves of species **S** at a point at which it occurs,” which concurs with the intention of Watson (1947). Wilson (2011) was incorrect, though, in attributing “cover repetition” to Greig‐Smith (1983), as Goodall discussed it along with “relative frequency,” sometimes confusingly called “repeated cover”. Goodall (1952) himself attributes “cover repetition” to Leonard Cockayne, an early New Zealand botanist, and applied it to naturally occurring species.

**Figure 1:**
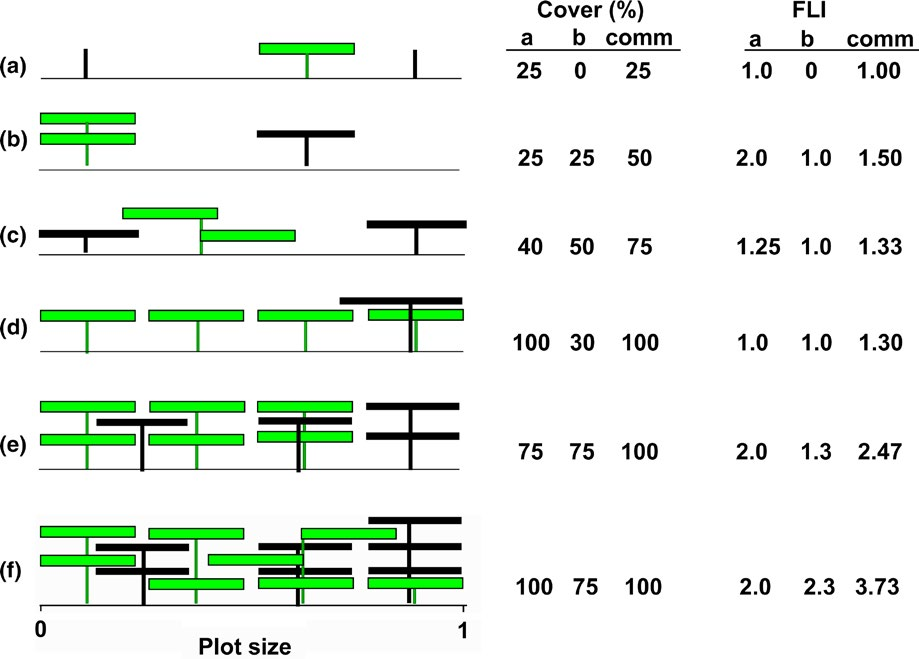
Comparison of two‐dimensional cover estimates of foliage (as the size of the shadow of each species or community at solar zenith), foliage layer index for each species (FLI), and FLI_comm_ for each of communities a–f, containing species *a* (green) and species *b* (black) expressed against a plot size of the horizontal line. Gaps between adjacent plants in the one stratum are exaggerated

Goodall's (1952) term, “cover repetition,” gives the classic meaning of LAI, for a single species, even if in a multispecies’ community, which is occasionally used in research with the approach of lowering pins into the canopy (e.g., O'Bryan, Prober, Lunt, & Eldridge, 2009; Southon, Green, Jones, Barker, & Power, 2012). At this point, it seems best to establish a more easily estimated concept under another term, for use by plant ecologists in a hurry. Foliage layer index (FLI) is a straightforward expression of the extent of self‐shading of a single species within a community (Fig. 1), calculated as:

$$\text{FLI}=\frac{\text{total foliage area of species}\;S}{\text{estimated cover of species}\;S\;\text{as a planar shadow.}}$$

Of course, the total foliage area of species **S** still has to be estimated, but this can be by any one of various methods, including destructively. And cover of **S** needs to be reported too. Mueller‐Dombois and Ellenberg (1974; p 60) describe an approach to estimating cover as “the vertical crown or shoot‐area projection per species in the plot.” An un‐misinterpretable (by students) explanation of this approach is the “size of the shadow of each species’ foliage at solar zenith” (e.g., Redpath & Rapson, 2015). Obviously, using the shadow approach, woody material, and photosynthetic petioles and stems etc. will all form part of the shadow and are included in the denominator by default. While in a review of the many ways in which cover values can be obtained, Wilson (2011) thoroughly criticizes such subjective methods, claiming these generate “nonsense measure[s],” Damgaard (2014) considers them unbiased at least, and Ónodi et al. (2017) found them reliable. Regardless, the subjective methods are so rapid and convenient that error in using them is accepted as the price of “doing business.”

As long as area and cover are both expressed in m^2^, then FLI is dimensionless. So if cover of species **S** is evaluated as % of a plot other than 1 m^2^, then it needs to be back‐converted into m^2^. This works for both a single individual of a species or for all the individuals of that species within a defined area. FLI also has the range ≥1, ecologically meaningful for a single species because auto‐competition can only start when the number of foliage layers is >1 (assuming the light source is vertically placed), and competition must be the ecological interest in defining a species’ positioning of its foliage.

**Figure 2:**
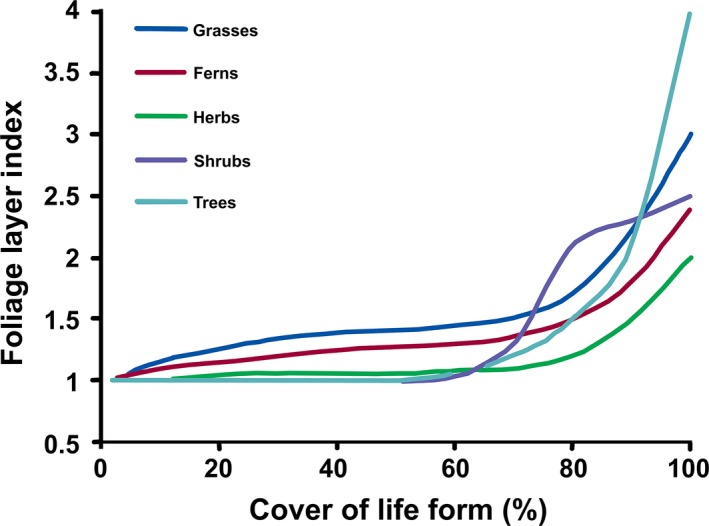
Hypothesized relationships between the number of layers of foliage a life‐form produces and cover of that life‐form in a plant community

What sort of results might FLI plausibly return in the field for any given species? Most angiosperms growing naturally, probably have a maximum FLI close to 1 anyhow, following the simplest model for making the best use of light (Figure 2). Herbs, especially rosette herbs, probably do not self‐shade much except at high density, because they have limited capacity for altering their leaf positions. Rhizomatous herbs are another matter. Grasses, notorious for a physical structure involving considerable self‐shading, often have large (>3) LAIs in crop situations, and probably in the natural world too, especially those grasses of tussock form (e.g., Mark, 1969). Responses are probably similar for ferns when tufted, as they typically are in New Zealand. Species with wood (even if we are discounting the role of branches in self‐shading—see Bréda, 2003; Wilson, 2011), tend to be older, and therefore taller, at higher covers. This opens them up to greater use of lateral or side light (i.e., reflected from outside the measurement zone, rather than incident solar radiation; Weiss, Baret, Smith, Jonckheere, & Coppin, 2004), and makes FLIs >> 1 less energetically wasteful, although FLI is correspondingly less sensible to measure. So shrubs should be able to develop higher FLIs. For broadleaved trees, taller and with a greater proportionate use of lateral or reflected light, maximum FLI should be yet higher. Overall FLIs should range from 1 to values of about 5–6, although some higher FLIs may well occur, especially in conjunction with phenotypic changes in leaf angles and Specific Leaf Areas. Conifers are more problematic though, as they generally have awl‐ or needle‐shaped leaves for which corrections are made in the global LAI database, which otherwise assumes leaves are one‐sided (Asner et al., 2003). However, an assumption of planarity or one‐sidedness is not necessary for a layer‐based measurement system, as any object can form a layer, even though that layer might be inefficient at intercepting incident solar radiation. In conifers, FLI values may well be very high. It remains to be seen if these predictions are plausible, and luckily there are enormous databases available for testing these suggestions.

Logically, LAI per community must be the sum of the FLIs per species scaled by some measure of a species’ abundance, such as cover; that is,

$${\text{FLI}}_{\mathrm{comm}}=\sum_{s=1}^{n}{\text{FLI}}_{s}*{\text{cover}}_{s}/{\text{cover}}_{\mathrm{comm}}$$

where *n* = the total number of species (s) in the community (comm), cover_s_ = % cover (by shadow at solar zenith) of species_s_ in the community, and cover_comm_ = total % cover of the community (= surface area of plot—area of bare ground). What sort of results might FLI_comm_ plausibly return in the field for any given community? Assuming that light is the limiting resource in any given environment, and evolution is efficient at producing diversity, then given the operational limits of seasonality and foliage turnover, most light should be consumed within a community (giving a high LAI). The number of foliage layers required to produce such a result possibly varys from one to eight (or thereabouts), depending on the light‐harvesting capacity of the individual species. Coniferous communities, very dark and often of low diversity, might only sustain only two or three species which generate eight foliage layers. Broadleaved deciduous forests might have 3‐5 layers of foliage, not necessarily all present at the same time. At the other end of the spectrum, chlorophyll‐rich species such as the New Zealand gully‐fern, *Blechnum colensoi*, might need only one layer to absorb most incident radiation (assuming no photo‐inhibition).

So to meaningfully describe the abundance (as an analogue for biomass) of a species within a community, determination of both cover per species and FLI is advisable. This addresses Wilson's (2011; Figure 2) criticism of “nonsense” cover estimates when totaled across a community, which occur precisely for the reason that the estimates do not incorporate differences in the placement of foliage within species, which should be called their FLI. It remains to be seen if these FLI_comm_ values are plausible.

## 5 CONCLUSION

FLI gives clear‐cut and easy to interpret values for foliage overlap within a species, even in multispecies’ communities (Figure 2), while LAI should be reserved for some estimate of density of light‐absorbing pigments per community. Crop scientists will need a mind‐shift here, but in nomenclature rather than methodology, while the larger body of remote‐sensing data is unaffected, although practitioners should consider whether they really are wanting to measure FLI_comm_!

## CONFLICT OF INTEREST

None declared.
